# Integrating external biological knowledge in the construction of regulatory networks from time-series expression data

**DOI:** 10.1186/1752-0509-6-101

**Published:** 2012-08-16

**Authors:** Kenneth Lo, Adrian E Raftery, Kenneth M Dombek, Jun Zhu, Eric E Schadt, Roger E Bumgarner, Ka Yee Yeung

**Affiliations:** 1 Department of Microbiology, University of Washington, Box 358070, Seattle, WA, 98195, USA; 2 Department of Statistics, University of Washington, Box 354320, Seattle, WA, 98195, USA; 3 Department of Biochemistry, University of Washington, Box 357350, Seattle, WA, 98195, USA; 4 Department of Genetics and Genomic Sciences, Mount Sinai School of Medicine, New York, NY, 10029, USA

**Keywords:** Systems biology, Network inference, Data integration, Statistics, Time-series expression data, Model uncertainty

## Abstract

**Background:**

Inference about regulatory networks from high-throughput genomics data is of great interest in systems biology. We present a Bayesian approach to infer gene regulatory networks from time series expression data by integrating various types of biological knowledge.

**Results:**

We formulate network construction as a series of variable selection problems and use linear regression to model the data. Our method summarizes additional data sources with an informative prior probability distribution over candidate regression models. We extend the Bayesian model averaging (BMA) variable selection method to select regulators in the regression framework. We summarize the external biological knowledge by an informative prior probability distribution over the candidate regression models.

**Conclusions:**

We demonstrate our method on simulated data and a set of time-series microarray experiments measuring the effect of a drug perturbation on gene expression levels, and show that it outperforms leading regression-based methods in the literature.

## Background

With recent advances in high-throughput biological data collection, reverse engineering of regulatory networks from large-scale genomics data has become a problem of broad interest to biologists. The construction of regulatory networks is essential for defining the interactions between genes and gene products, and predictive models may be used to develop novel therapies [1,2]. Both microarrays and more recently next generation sequencing provide the ability to quantify the expression levels of all genes in a given genome. Often, in such experiments, gene expression is measured in response to drug treatment, environmental perturbations, or gene knockouts, either at steady state or over a series of time points. This type of data captures information about the effect of one gene’s expression level on the expression level of another gene. Hence, such data can, in principle, be reverse engineered to provide a regulatory network that models these effects.

A regulatory network can be represented as a directed graph, in which each node represents a gene (in our case an mRNA level) and each directed edge (*r→g*) represents the relationship between regulator *r* and gene *g*. We aim to infer the directed edges that describe the relationships among the nodes. In this case, the causal relationship is statistically inferred, in contrast to the classic definition of causality used in biology to imply direct physical interaction leading to a phenotypic change. This is a challenging problem, especially on a genome-wide scale, since the goal is to unravel a small number of regulators (parent nodes) out of thousands of candidate nodes in the graph. Even with high-dimensional gene expression data, network inference is difficult, in part because of the small number of observations for each gene. In order to improve network inference, one would like a coherent approach to integrate external knowledge and data to both fill in gaps in the gene expression data and to constrain or guide the network search.

In this article, we present a network inference method that addresses the dimensionality challenge with a Bayesian variable selection method. Our method uses a supervised learning framework to incorporate external data sources. We applied our method to a set of time-series mRNA expression profiles for 95 yeast segregants and their parental strains, over six time points in response to a drug perturbation. This extends our previous work [3] by incorporating prior probabilities of transcriptional regulation inferred using external data sources. Our method also accommodates feedback loops, a feature allowed only in some current network construction methods.

### Previous work

Bayesian networks [4-6] are one of the most popular modeling approaches for network construction using gene expression data [7-17]. A Bayesian network is a probabilistic graphical model for which the joint distribution of all the nodes is factorized into independent conditional distributions of each node given its parents. The goal of Bayesian network inference is to arrive at a directed graph such that the joint probability distribution is optimized globally. While different Bayesian network structures may give rise to the same probability distribution, so that such networks in general do not imply causal relationships, prior information can be used to break this nonidentifiability so that causal inferences can be made. For example, systematic sources of perturbation such as naturally occurring genetic variation in a population or specific drug perturbations in which response is observed over time can lead to reliable causal inference [1,2,18,19]. A Bayesian network is a directed acyclic graph (DAG). Therefore, cyclic components or feedback loops cannot be accommodated. This DAG constraint is an obstacle to using the Bayesian network approach for modeling gene regulatory networks because feedback loops are typical in many biological systems [20]. The DAG constraint is removed when dynamic Bayesian networks are used to model time-series expression data [19,21-24]. Dynamic Bayesian networks represent genes at successive time points as separate nodes, thus allowing for the existence of cycles. Bayesian network construction is an NP-hard problem [25,26], with computational complexity increasing exponentially with the number of nodes considered in the network construction process. In spite of some attempts to reduce the computational cost [27], the Bayesian network approach in general is computationally intensive to implement, especially for network inference on a genome-wide scale.

In regression-based methods, network construction is recast as a series of variable selection problems to infer regulators for each gene. The greatest challenge is the fact that there are usually far more candidate regulators than observations for each gene. Some authors have used singular value decompositions to regularize the regression models [28-30]. Others have built a regression tree for each target gene, using a compact set of regulators at each node [31-34]. Huang et al. [35] used regression with forward selection after pre-filtering of candidates deemed irrelevant to the target gene, and Imoto et al. [16] used nonparametric regression embedded within a Bayesian network. *L*1-norm regularization, including the elastic net [36,37] and weighted LASSO [38], has also been widely used [39-49].

Ordinary differential equations (ODE) provide another class of network construction strategies [50-53]. Using first-order ODEs, the rate of change in transcription for a target gene is described as a function of the expression of its regulators and the effects caused by applied perturbations. ODE-based methods can be broadly classified into two categories, depending on whether the gene expressions are measured at steady state [54-58] or over time [51-53]. As an example, the TSNI (Time Series Network Identification) algorithm used ODEs to model time series expression data subject to an external perturbation [53]. To handle the dimensionality challenge (i.e. the number of observations per gene is much smaller than the number of genes), Bansal et al. employed a cubic smoothing spline to interpolate additional data points, and applied Principal Component Analysis to reduce dimensionality.

To help mitigate problems with using gene expression data in network inference, external data sources can be integrated into the inference process. Public data repositories provide a rich resource of biological knowledge relevant to transcriptional regulation. Integrating such external data sources into network inference has become an important problem in systems biology. James et al. [43] incorporated documented experimental evidence about the presence of a binding site for each known transcription factor (TF) in the promoter region of its target gene in *Escherichia coli*. Djebbari and Quackenbush [13] used preliminary networks derived from literature indexed in PubMed and protein-protein interaction (PPI) databases as seeds for their Bayesian network analysis. Zhu et al. [59] showed that combining information from TF binding sites and PPI data increased overall predictive power. Geier et al. [15] examined the impact of external knowledge with different levels of accuracy on network inference, albeit on a simulated setting. Imoto et al. [16] described different ways to specify knowledge about PPI, documented regulatory relationships and well-studied pathways as prior information. Lee et al. [44] presented a systematic way to include various types of biological knowledge, including the gene ontology (GO) database, ChIP-chip binding experiments and a compressive collection of information about sequence polymorphisms.

### Our contributions

This article is an extension of Yeung et al. [3] which adopted a regression-based framework in which candidate regulators are inferred for each gene using expression data at the previous time point. Iterative Bayesian model averaging (iBMA) [60-62] was used to account for model uncertainty in the regression models. A supervised framework was used to estimate the relative contribution of each type of external knowledge and from this a shortlist of promising regulators for each gene was predicted. This shortlist was used to infer regulators for each gene in the regression framework.

**Figure 1:**
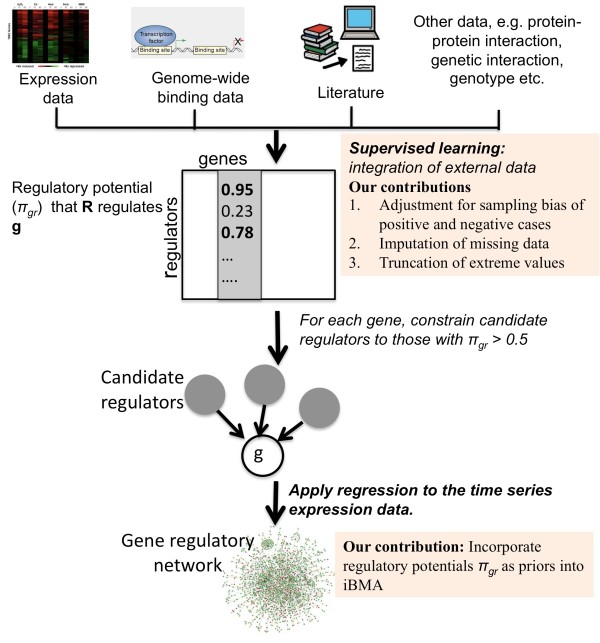
Overview of iBMA-prior with a highlight of our main contributions.

Our contributions are four-fold. First, we develop a new method called iBMA-prior that explicitly incorporates external biological knowledge into iBMA in the form of a prior distribution. Intuitively, we consider models consisting of candidate regulators supported by considerable external evidence to be frontrunners. A model that contains many candidate regulators with little support from external knowledge is penalized. Second, we demonstrate the merits of specifying the expected number of regulators per gene as priors through iBMA-size, which is a simplified version of iBMA-prior without using gene-specific external knowledge. Third, we refine the supervised framework to adjust for sampling bias towards positive cases in the training data, thereby calibrating the prior distribution. Fourth, we expand our benchmark to include simulated data, and compare our iBMA methods to L1-regularized regression-based methods. Specifically, we applied iBMA-prior to real and simulated time-series gene expression data, and found that it out-performed our previous work [3] and other leading methods in the literature on these data, producing more compact and accurate networks. Figure 1 summarizes iBMA-prior and our main contributions.

## Results and discussion

We applied our method, iBMA-prior, to a time-series data set of gene expression levels for 95 genotyped haploid yeast segregants perturbed with the macrolide drug rapamycin over 6 time points [3]. These data are described in detail in the *Methods* section. To evaluate the performance of iBMA-prior, other published regression-based network construction methods were applied to the same time-series gene expression data set and the resulting networks were assessed for the recovery of documented regulatory relationships that were not used in the network construction process. We also checked whether each method recovered target genes enriched in upstream regions containing the binding sites of known TFs. We further carried out a simulation study to assess our method.

### Comparison of different methods

First, we assessed the improvement of iBMA-prior over that of our previous work iBMA-shortlist from Yeung et al. [3] (see Methods for details) when applied to the same yeast time-series gene expression data. Then, we compared our BMA-based methods to several L1-regularized methods, including the least absolute shrinkage and selection operator (LASSO) [36,63] and least angle regression (LAR) [64]. Regularized regression methods combine shrinkage and variable selection. L1-regularized methods aim to minimize the sum of squared errors with a bound on the sum of the absolute values of the coefficients [65]. Efficient implementations are available for some of these methods, including LASSO and LAR, and these methods have been applied to high-dimensional data in which there are more variables than observations [64,66,67].

We also compared the performance of our method with and without using external biological knowledge. We assessed hybrid methods by combining LASSO and LAR with the same supervised learning stage that was used in iBMA-prior and iBMA-shortlist. Table 1 lists all the methods compared in this analysis.

**Table 1:** Different regression-based methods applied to the time-series gene expression data to construct gene regulatory networks

**Method**	**Data used**	**Description**
iBMA-prior	Gene expression + external data	Our proposed methodology that incorporates prior model probabilities in BMA. These prior probabilities were computed using external data sources.
iBMA-shortlist	Gene expression + external data	Iterative BMA that uses external knowledge to shortlist *p* = 100 candidates for each target gene. The revised supervised step was used. Unlike iBMA-prior, the information from the external data is not used in variable selection via BMA.
Network A from Yeung et al. [3]	Gene expression + external data	This method is the same as in iBMA-shortlist, but using the old version of supervised step described in Yeung et al. [3]. We aim to study the impact of the revised supervised step by comparing iBMA-shortlist to network A.
LASSO-shortlist	Gene expression + external data	LASSO [36,63] with the use of external knowledge to shortlist *p* = 100 candidates for each target gene.
LAR-shortlist	Gene expression + external data	LAR [64] with the use of external knowledge to shortlist *p* = 100 candidates for each target gene.
iBMA-size	Gene expression data only	A simplified version of iBMA-prior that disregards external knowledge, except for setting *π*_*gr*_ = *τ* = 2.76/6000 = 0.00046 for all *g* and *r*. This essentially turns Eq. (5) into a function of model size only.
iBMA-noprior	Gene expression data only	Iterative BMA without any use of external knowledge.
LASSO-noprior	Gene expression data only	LASSO without any use of external knowledge.
LAR-noprior	Gene expression data only	LAR without any use of external knowledge.

### Assessment: recovery of documented relationships

To evaluate the accuracy of the network constructed by each method, we assessed its concordance with the Yeastract database, a curated repository of regulatory relationships between known TFs and target genes in the *Saccharomyces cerevisiae* literature [68]. If a regulatory relationship documented in Yeastract was also inferred in the network, we concluded that this relationship was recovered by direct evidence. Some of the positive examples used in the supervised learning stage are also documented in Yeastract. To avoid bias, we did not consider those regulatory relationships in the assessment. For each method compared, we applied Pearson’s chi-square test to a 2 × 2 contingency table that quantified the concordance of the inferred network with the Yeastract database. We also computed the true positive rate (TPR), defined as the proportion of the inferred positive relationships that are documented in Yeastract. It should be noted that Yeastract cannot document all “true” relationships as the entire set of regulatory relationships in yeast has yet to be defined. We further considered the ratio of the observed number of recovered relationships to its expected count as a result of random assortment (O/E). More detailed definitions of the assessment criteria can be found in Additional file 1: Figure S1.

Table 2 summarizes the assessment results for the nine methods compared. Additional details are presented in Additional file 2: Table S1. First, we studied the impact of integrating external knowledge into the network construction process under the iBMA framework. The TPR of iBMA-prior was 18.00%, and the number of recovered positive relationships was 593, which is 4.11 times more than the expected number by random chance. Using the revised supervised step described in this work without incorporating prior probabilities into the iBMA framework, iBMA-shortlist yielded a TPR of 12.78% and O/E ratio of 2.92. This is an improvement over network A (TPR = 9.98% and O/E = 2.28) constructed using the same algorithm and our previous version of the supervised framework as described in Yeung et al. [3]. All of our methods that incorporate external knowledge (iBMA-prior, iBMA-shortlist and network A) produced higher TPRs than iBMA-noprior for which only the time-series gene expression data were used. In particular, iBMA-prior produced a TPR (18.00%), which represents a two-fold increase over iBMA-noprior (8.9%). Therefore, the integration of external data clearly improved the recovery of known relationships, and our latest method, iBMA-prior, performed the best.

**Table 2:** Summary of the assessment result for different network construction methods on the time-series gene expression data

**Method**	**Data used**	**Network size**	***p*-value of chi sq test**^***a***^	**TPR (%)**^***b***^	**# mis-class.**^***c***^	**TP**	**O/E**^***d***^
iBMA-prior	Gene expression + external data	21951	<1.00E-320	18.00	19282	593	4.11
iBMA-shortlist	Gene expression + external data	67440	<1.00E-320	12.78	24673	1287	2.92
Network A from Yeung et al.	Gene expression + external data	65122	1.68E-111	9.98	22485	662	2.28
LASSO-shortlist	Gene expression + external data	255293	<1.00E-320	11.07	46482	4169	2.53
LAR-shortlist	Gene expression + external data	242495	<1.00E-320	11.28	44765	4017	2.57
iBMA-size	Gene expression data only	17202	5.75E-56	16.84	17622	114	3.84
iBMA-noprior	Gene expression data only	63026	1.75E-23	8.85	18903	186	2.02
LASSO-noprior	Gene expression data only	564321	2.56E-10	5.20	38399	1231	1.19
LAR-noprior	Gene expression data only	194687	1.38E-40	7.71	22777	511	1.76

^*a*^ The *p*-value of Pearson’s chi-square test measures the strength of association between an inferred network and the Yeastract database.

^*b*^ True positive rate (TPR) is defined as the proportion of inferred regulatory relationships that are documented in Yeastract.

^*c*^ The number of misclassified cases is the sum of false positives and false negatives.

^*d*^ The O/E ratio is the number of folds the observed number of recovered relationships (i.e., TP) in excess of the expected count of recovery by chance.

Next, we compared our iBMA-based methods to L1-regularized methods. All the approaches that used LASSO and LAR generated networks that had far more mis-classifications than the iBMA-based methods. Specifically, applications of LASSO or LAR without the supervised framework (LASSO-noprior and LAR-noprior) had TPRs of 5.20% and 7.71% respectively, the lowest among all the methods considered. Incorporating external knowledge did improve both LASSO and LAR, increasing the TPRs to about 11% in both LASSO-shortlist and LAR-shortlist. However, these TPRs were still lower than the TPRs for our iBMA-based methods. Our iBMA-based methods therefore outperformed methods based on LASSO and LAR for these data.

Finally, we investigated the impact of priors in iBMA-size, in which we applied a model size prior to calibrate the sparsity of the inferred networks without using any external data sources. iBMA-size can be considered as a simplified version of iBMA-prior that sets the regulatory potential (the prior probability that a candidate regulates a given gene) to a constant parameter that controls the expected number of regulators per gene. From Table 2, iBMA-size produced a TPR of 16.84%, which was higher than all the other methods considered except iBMA-prior. Although the number of recovered positive relationships was lower than that of iBMA-prior (114 <593), iBMA-size also produced a network that was more compact (17,202 edges compared to 21,951 edges). We would recommend iBMA-size when gene-specific external information is not available.

In Table 2 and Additional file 2: Table S1, all the iBMA networks were thresholded at a posterior probability of 50% (i.e., edges with posterior probability <50% were removed). We found that iBMA-prior also out-performed other methods for these data over different posterior probability thresholds (see Additional file 2: Table S2).

### Assessment: transcription factor binding site analysis

In another assessment, we checked whether the set of target genes containing known binding sites for a certain TF were enriched among the child nodes of that TF in each inferred network. We first extracted the known binding sites for 129 TFs documented in the JASPAR database [69,70]. Using TFMscan [71], we retrieved a set of genes containing the known binding sites in their upstream regions for each TF. We then checked for enrichment of these genes among the inferred child nodes of the corresponding TFs in each network with Fisher’s exact test. Table 3 reports the number of TFs whose inferred child nodes exhibited such enrichment, at a false discovery rate (FDR) of 10%. All of the methods that made use of external information outperformed all of those that did not, illustrating the benefit of incorporating external knowledge. LASSO-shortlist and LAR-shortlist appeared to produce slightly better results than iBMA-prior in this binding site analysis, but it is likely the consequence of their larger network sizes (>2x larger than iBMA prior).

**Table 3:** Number of transcription factors with gene sets containing their known binding sites enriched by the different methods in comparison

**Method**	**Data used**	**# TFs with enriched gene sets**^***a***^
iBMA-prior	Gene expression + external data	38
iBMA-shortlist	Gene expression + external data	30
LASSO-shortlist	Gene expression + external data	41
LAR-shortlist	Gene expression + external data	44
iBMA-size	Gene expression data only	4
iBMA-noprior	Gene expression data only	9
LASSO-noprior	Gene expression data only	13
LAR-noprior	Gene expression data only	10

^*a*^ FDR was controlled at 10%.

### Comparison with Lirnet

Lee et al. [44] proposed a regression-based network construction method called Lirnet, which performed well on a publicly available gene expression data set from Brem et al. [72]. The Brem data set recorded the steady-state expression levels for 112 yeast segregants, 95 of which were profiled in our time-series experiments under different growth conditions. Lee et al. [44] showed that Lirnet out-performed Bayesian networks on the same data, and so we compared our top performer, iBMA-prior, with Lirnet. Because Lirnet was formulated to analyze steady-state expression data with no time components, we adapted our method to static data by removing the subscript referring to the time point from Equation (4):

(1) $$E[X_{g,s}|D]=\beta_{g,0}+\sum_{r\in R_{g}}\beta_{g,r}X_{r,s},$$

We applied iBMA-prior to the same 3152-gene subset of the Brem et al. data that Lee et al. [44] used. Lirnet constrained the search of regulators for each target gene to 304 known TFs. For fair comparison, we also confined the set of candidate regulators to the same TFs. Networks constructed from steady-state gene expression data cannot have feedback loops [73-75]. To detect and remove such loops from our inferred network, we identified all strongly connected components using the igraph R package, and deleted the TF-gene link associated with the lowest posterior probability for each cycle.

Same as before, we evaluated different methods by assessing the concordance of the inferred networks with the Yeastract database using Pearson’s chi-square test. The assessment results in Table 4 show that iBMA-prior outperformed Lirnet, almost doubling the TPR and the O/E ratio while producing a comparable number of misclassified regulatory relationships.

**Table 4:** Comparison of iBMA-prior, iBMA-shortlist and Lirnet in network construction on the Brem data

**Method**	**Network size**	***p*-value of chi square test**^***a***^	**TPR (%)**^***b***^	**# misclass.**^***c***^	**TP**	**O/E**^***d***^
iBMA-prior	8000	7.75E-65	15.62	10198	323	2.41
iBMA-shortlist	35995	1.02E-59	10.99	14581	818	1.70
Lirnet	10491	1.90E-03	8.42	10080	132	1.30

^*a*^ The *p*-value of Pearson’s chi-square test measures the strength of association between an inferred network and the Yeastract database.

^*b*^ True positive rate (TPR) is defined as the proportion of inferred regulatory relationships that are documented in Yeastract.

^*c*^ The number of misclassified cases is the sum of false positives and false negatives.

^*d*^ The O/E ratio is the number of folds the observed number of recovered relationships (i.e., TP) in excess of the expected count of recovery by chance.

### Simulation study

We designed and conducted a series of simulations to further assess our proposed method. We used the fitted model obtained from applying iBMA-prior to the yeast time-series microarray data set as the true underlying network, and generated simulated expression data from the estimated linear regression model. Twenty data sets, each with the same dimensions as the real time-series expression data, were independently generated as follows:

1. Set the prior probability of a regulatory relationship for each gene pair to the same value as the regulatory potential obtained at the supervised learning stage using the real external data.

2. Set the expression levels of the 3556 genes for the 95 yeast segregants and the two parental strains at time *t* = 0 as the observed measurements in the real yeast time-series gene expression data.

3. For each target gene *g*, define the set *R*_*g*_ of true regulators as those with a posterior probability of ≥50% in our inferred network using iBMA-prior and the real time-series data.

4. For time *t* = 1 to 5,

For gene *g* = 1 to 3556, generate the simulated true expression level for each segregant *s* using the following equation:

(2) $$X_{g,t,s}^{\text{true}}=\beta_{g,0}+\sum_{r\in R_{g}}\beta_{g,r}X_{r,t-1,s}^{\text{true}},$$

where the *β*’s are given by the posterior expectation of the regression coefficients corresponding to the set of true regulators determined in Step 3.

5. Generate the simulated observed gene expression levels by adding noise to the true expression levels without measurement errors, i.e.,

(3) $$X_{g,t,s}=X_{g,t,s}^{\text{true}}+\epsilon_{g,t,s},$$

where *ϵ*_*g*,*t*,*s*_ ~ N(0, *σ*_*g*_^2^) with *σ*_*g*_^2^ being given by the sample variance of the regression residuals in the real data analysis. Others, e.g. [76], have shown that the error in log ratios of expression data is reasonably approximately by a normal distribution.

To assess the accuracy of networks inferred with the simulated data sets, we compared each of these networks to the true network created in Step 3 of the data generation algorithm. We used the same assessment criteria as in the real data analysis with the true network replacing Yeastract as the reference. As shown in Table 5, iBMA-prior out-performed the other iBMA-based methods, yielding a TPR of 71.13% averaged over 20 replications (compared to 47.23% for iBMA-shortlist, 20.31% for iBMA-size, and 8.55% for iBMA-noprior).

**Table 5:** Assessment result for the different methods applied to data sets generated in the stimulation study

**Method**	**Data used**	**Network size**	***p*-value of chi sq test**^***a***^	**TPR (%)**^***b***^	**# mis-class.**^***c***^	**TP**
iBMA-prior	Generated data + prior probability matrix	14011	<1.00E-320	71.13	16029	9966
iBMA-shortlist	Generated data + prior probability matrix	30753	<1.00E-320	47.23	23652	14526
iBMA-size	Generated data only	9349	<1.00E-320	20.31	27503	1899
iBMA-noprior	Generated data only	29393	<1.00E-320	8.55	46317	2513

^*a*^ The *p*-value of Pearson’s chi-square test measures the strength of association between an inferred network and the true network for the simulation study.

^*b*^ True positive rate (TPR) is defined as the proportion of correctly inferred regulatory relationships.

^*c*^ The number of misclassified cases is the sum of false positives and false negatives.

Remark: The values reported in the table were averaged across the 20 replications. The true network for the simulation study contained a total of 21951 edges.

## Conclusions

In this article, we have proposed a methodology that systematically integrates external biological knowledge into BMA for network construction. A key feature of our approach is a formal mechanism to account for model uncertainty. For each target gene, we arrive at a compact set of promising models from which to draw inference, the weights of which are calibrated by the external biological knowledge. Our method infers sparse, compact and accurate networks upon the input of a reasonable estimate of network density from both real and simulated data. It does not put a hard limit on the number of regulators per target gene, unlike some other methods, such as Bayesian network approaches that impose this constraint to reduce the computational burden. While known TFs are in general favored *a priori* with the available external biological knowledge, we do not confine the search for regulators to them. This allows for the discovery of new regulatory relationships.

We showed that our method, iBMA-prior, consistently outperformed our previous method [3] using both real and simulated time-series gene expression data. We showed that this improvement is mostly due to the incorporation of external data sources via prior probabilities (iBMA-prior versus iBMA-shortlist in Table 2). We also improved upon our previous supervised method by adjusting for the sampling bias of positive and negative training samples (iBMA-shortlist versus network A in Table 2). We further showed that our iBMA-based methods (iBMA-prior and iBMA-shortlist) recovered a higher percentage of known regulatory relationships (i.e. higher TPRs) than other popular variable selection methods (LASSO and LAR).

A key contribution of this work is the derivation of more compact networks with higher TPRs. Unfortunately, due to incomplete knowledge, the evaluation of false positives and false negatives is difficult using real data. Therefore, we supplemented our study with a simulation study designed to mimic the real data, and showed that iBMA-prior produced fewer misclassified cases (i.e. the sum of false positives and false negatives) than other iBMA-based methods.

There are many directions for future work. A time-lag regression model, i.e., one that accounts for the current expression level of a target gene with the past expression levels of its regulators, is used in our methodology. This model formulation is in line with many other regression-based methods targeting time-series gene expression data [3,28,35,48,49]. The expression levels were taken at regular time intervals in our yeast time-series gene expression data set. If the levels were measured at non-uniform time intervals, we could create interpolated time-series data with interpolation strategies employed in the literature [51,53]. It would be useful to apply our methodology to network construction in prokaryotic systems as we would expect better performance in these less complex systems that tend to be more dominated by transcriptional control [77].

## Methods

### Time-series gene expression data for yeast segregants

We applied our method to a set of time-series mRNA expression data measuring the gene expression levels of 95 genotyped haploid yeast segregants perturbed with the macrolide drug rapamycin [3]. These segregants, along with their genetically diverse parents, BY4716 (BY) and RM11-1a (RM), have been genotyped previously [72]. Rapamycin was chosen for perturbation because it was expected to induce widespread changes in global transcription, based on a screen of the public microarray data repositories [78-80]. This perturbation allowed for the capture of a large subset of all regulatory interactions encoded by the yeast genome. Each yeast culture was sampled at 10-minute intervals for 50 minutes after rapamycin addition. The RNA purified from these samples was profiled with Affymetrix Yeast 2.0 microarrays. Probe signals were summarized into gene expression levels using the Robust Multi-array Average (RMA) method [81] and genes not exhibiting significant changes in expression were filtered from the data as described in [3]. The data subset that remained consisted of the time-dependent mRNA expression profiles of 3556 genes. The complete time series gene expression data are publicly available at ArrayExpress (http://www.ebi.ac.uk/arrayexpress/) with accession number E-MTAB-412.

### Bayesian model averaging (BMA)

BMA is a variable selection approach that takes model uncertainty into account by averaging over the posterior distribution of a quantity of interest based on multiple models, weighted by their posterior model probabilities [82,83]. In BMA, the posterior distribution of a quantity of interest Θ given the data *D* is given by $\text{Pr}\left(\Theta |D\right)=\sum_{k=1}^{K}\text{Pr}\left(\Theta |D,M_{k}\right)\text{Pr}\left(M_{k}|D\right)$, where *M*_1_,…,*M*_*k*_ are the models considered. Each model consists of a set of candidate regulators. In order to efficiently identify a compact set of promising models *M*_*k*_ out of all possible models, two approaches are sequentially applied. First, the leaps and bounds algorithm [84] is applied to identify the best *nbest* models for each number of variables (i.e., regulators). Next, Occam’s window is applied to discard models with much lower posterior model probabilities than the best one [85]. The Bayesian Information Criterion (BIC) [86] is used to approximate each model's integrated likelihood, from which its posterior model probability can be determined.

While BMA has performed well in many applications [60], it is hard to apply directly to the current data set in which there are many more variables than samples. Yeung et al. [62] proposed an iterative version of BMA (iBMA) to resolve this problem. At each iteration, BMA is applied to a small number, say, *w* = 30, of variables that could be efficiently enumerated by leaps and bounds. Candidate predictor variables with a low posterior inclusion probability are discarded, leaving room for other variables in the candidate list to be considered in subsequent iterations. This procedure continues until all the variables have been processed.

### Supervised framework for the integration of external knowledge

We formulated network construction from time series data as a regression problem in which the expression of each gene is predicted by a linear combination of the expression of candidate regulators at the previous time point. Let *D* be the entire data set and *X*_*g*,*t*,*s*_ be the expression of gene *g* at time *t* in segregant *s*. Denote by *R*_*g*_ the set of regulators for gene *g* in a candidate model. The expression of gene *g* is formulated by the following regression model:

(4) $$E[X_{g,t,s}|D]=\beta_{g,0}+\sum_{r\in R_{g}}\beta_{g,r}X_{r,t-1,s},$$

where *E* denotes expectation and *β*’s are regression coefficients. For each gene, we apply iBMA to infer the set of regulators.

To account for external knowledge in the network construction process, Yeung et al. [3] introduced a supervised framework to estimate the weights of various types of evidence of transcriptional regulation and subsequently derived top candidate regulators. For instance, a target gene is likely to be co-expressed with its regulators across diverse conditions in publicly available, large-scale microarray experiments [78,87,88]. ChIP-chip data [89] provide supporting evidence for a direct regulatory relationship between a given TF and a gene of interest by showing that the TF directly binds to the promoter of that gene. A candidate regulator with known regulatory roles in curated databases such as the *Saccharomyces* Genome Database (SGD) [90] would be favored *a priori*. Polymorphisms in the amino acid sequence of a candidate regulator that affect its regulatory potential provide further evidence of a regulatory relationship [44]. Common gene ontology (GO) [91] annotations for a target gene and candidate regulators also provide evidence of functional relationship.

To study the relative importance of the various types of external knowledge from the supervised framework, we collected 583 positive examples of known regulatory relationships between TFs and target genes from the *Saccharomyces cerevisiae* Promoter Database (SCPD) [92] and the Yeast Protein Database (YPD) [93]. Random sampling of these TF-gene pairs was used to generate 444 negative examples. Logistic regression using BMA was applied to estimate the contribution of each type of external knowledge in the prediction of regulatory relationships. The fitted model was then used to predict the regulatory potential *π*_*gr*_ of a candidate regulator *r* for a gene *g*, i.e., the prior probability that candidate *r* regulates gene *g*, for all possible regulator-gene pairs. Next, the regulatory potentials were used to rank and shortlist the top *p* candidate regulators for each gene (*p* = 100 by default in our experiments). The shortlisted candidates were then input to BMA for variable selection in the network construction process.

### Incorporating prior probabilities into iBMA

The potential benefit of using information from external knowledge to refine the search for regulators was shown by Yeung et al. and many others [3,13,15-17,43,44]. However, external knowledge was only used to shortlist the top *p* candidate regulators for each target gene in Yeung et al. Here, we develop a formal framework that fully incorporates external knowledge into the BMA network construction process.

We associate each candidate model *M*_*k*_ with a prior probability, namely:

(5) $$\text{Pr}\left(M_{k}\right)\propto \prod_{r}\pi_{\text{gr}}^{\delta_{\text{kr}}}{\left(1-\pi_{\text{gr}}\right)}^{1-\delta_{\text{kr}}},$$

where *π*_*gr*_ is the regulatory potential of a candidate regulator *r* for a gene *g, δ*_*kr*_ = 1 if *r* ∈*M*_*k*_ and *δ*_*kr*_ = 0 otherwise [85,94]. Intuitively, we consider models consisting of candidate regulators supported by considerable external evidence to be frontrunners. A model that contains many candidate regulators with little support from external knowledge is penalized.

The posterior model probability of model *M*_*k*_ is given by

(6) $$\text{Pr}\left(M_{k}|D\right)\propto f\left(D|M_{k}\right)\text{Pr}\left(M_{k}\right),$$

where *f*(*D* | *M*_*k*_) is the integrated likelihood of the data *D* under model *M*_*k*_, and the proportionality constant ensures that the posterior model probabilities sum up to 1.

Then Occam’s window was used to discard any model *M*_*k*_ having a posterior odds less than 1/*OR* relative to the model with the highest posterior probability, *M*_*opt*_. The parameter *OR* controls the compactness of the set of selected models, and here we set it to 20.

### Extension of iBMA: cumulative model support

In Yeung et al. [3], the models selected in an intermediate iteration by iBMA were not recorded once that iteration was completed, and the final set of models selected were chosen only from those considered in the last iteration. While computationally efficient, this strategy overlooked the possibility of accumulated model support over multiple iterations. We improve the model selection process by storing all the models selected in any iteration and applying Occam’s window to this cumulative set of models as the last step in the algorithm.

At the end of each iteration of iBMA, and after applying Occam’s window to all models considered, we compute the posterior inclusion probabilities for each candidate regulator *r* by summing up the posterior probabilities of all models that involve this regulator.

(7) $$\text{Pr}(\beta_{\text{gr}}\neq 0|D)=\sum_{k:M_{k}\in \Phi }\text{Pr}\left(M_{k}|D\right)\cdot \delta_{\text{kr}}\text{.}$$

where F is the set of all possible models for gene g, β_*gr*_ is the regression coefficient of a candidate regulator *r* for a gene *g, δ*_*kr*_ = 1 if *r* ∈*M*_*k*_ and *δ*_*kr*_ = 0 otherwise. Finally, we infer regulators for each target gene *g* by thresholding on the posterior inclusion probability at a predetermined level (50% in all our experiments unless otherwise specified).

### Extensions of the supervised framework

We have extended the supervised framework of Yeung et al. [3] in three ways.

#### Imputation of missing values in ChIP-chip data

About 9% of the ChIP-chip data used in the training samples were originally undefined. The ChIP-chip data take the form of *p*-values for the statistical tests of whether candidate regulator *r* binds to the upstream region of gene *g in-vivo*. In [3], those undefined values were regarded as lack of evidence for upstream binding and assigned values of one. Here, we used multiple imputation [95,96], in which we sampled with replacement from the empirical distribution of the non-missing ChIP-chip data, conditioning on the presence or absence of regulatory relationships. We used 20 imputations as recommended by Graham et al. [97] for scenarios with about 10% missing data. Logistic regression was then performed on the training sample filled with the imputed ChIP-chip values.

#### Truncation of extreme values in external data

Some of the external data types used in the supervised learning stage contained value ranges for individual genes that far exceeded the ranges for these genes in the training samples, e.g. the SNP-level information in Additional file 2: Table S3. Therefore, we truncated all extreme values in the external data to the respective maximum value observed in the training samples.

#### Adjustment for sampling bias regarding positive and negative cases

**Figure 2:**
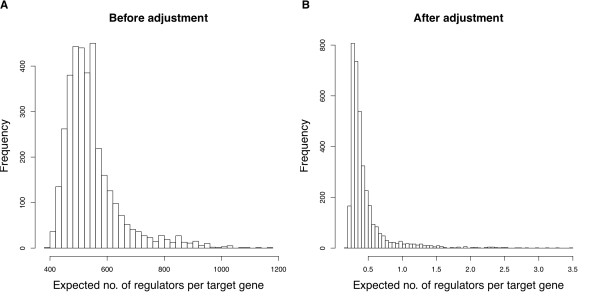
**The expected number of regulators per target gene in accordance with external knowledge.** Histogram of the expected number of regulators per target gene in the **A**. absence / **B**. presence of a proper measure to account for the difference in sampling rates for positive and negative examples respectively at the supervised learning stage.

In the supervised framework of Yeung et al., the expected number of regulators per target gene, computed as the sum of regulatory potentials of all candidate regulators, mostly fell between 400 and 600 (see Figure 2(a)). Such an apparent overestimation of positive regulatory relationships was due to the fact that similar numbers of positive and negative examples in the supervised learning stage. Given the sparse nature of a gene regulatory network, we expect the number of TF-gene pairs with regulatory relationships to be a small proportion of the total.

Here, we address this issue by using a strategy that is commonly used in case–control studies, in which disease (positive) cases are usually rare [98,99]. Let *π*_1_ and *π*_0_ be the sampling rates for positive and negative cases respectively. To adjust for the difference in the sampling rates, we add an offset of -log(*π*_1_/*π*_0_) to the logistic regression model. Equivalently, we divide the predicted odds by *π*_1_/*π*_0_. Previous literature has suggested that the in-degree distribution of gene regulatory networks decays exponentially [100-102]. Based on regulatory relationships documented in various yeast databases [90,92,93,103,104], Guelzim et al. [100] empirically estimated the in-degree distribution of the regulatory network as 157*e*^-0.45*m*^, where *m* denotes the number of TFs for a target gene. This implies that each target gene is regulated by approximately 2.76 TFs on average. Since we have 583 positive training examples, 444 negative examples, and 6000 yeast genes, we characterize such a network with density *τ* = 2.76/6000 = 0.00046, and compute $\pi_{1}=\frac{583}{6000\times 2.76}=3.52\%$, and $\pi_{0}=\frac{444}{\left[6000\times \left(6000-2.76\right)\right]}=0.0012\%$. Therefore, we divide all the predicted odds by *π*_1_/*π*_0_ = 2853. For instance, if the original predicted probability is 0.9, i.e., the predicted odds is 9, then after scaling the odds adjusted for sampling bias, it becomes 9/2853 = 0.0032, implying an adjusted probability of 0.0032. As shown in Figure 2(b), the expected number of regulators per target gene has dropped substantially to a level of around 0.5 after our three correction strategies (adjustment of sampling bias, imputation of missing ChIP-chip values and truncation of extreme values) are applied. Additional file 1: Figure S2 shows the incremental merit of our correction strategies. Additional file 2: Table S3 gives the estimated regression coefficient and the posterior probability for each external data type in our revised supervised framework.

To assess the sensitivity of our results to changes in the assumed prior average number of regulators per target gene, we repeated the analysis with various levels of the network density *τ*, and found that the assessment results were comparable. Please see the Additional file 3 for complete details.

### Summary: outline of algorithm

1. For each gene *g*, rank the candidate regulators based on the regulatory potentials predicted from the supervised framework.

2. Shortlist the top *p* candidates from the ranked list (*p* = 100 in our experiments).

3. Fill the BMA window with the top *w* candidates in the shortlist (*w* = 30 in our experiments).

4. Apply BMA with prior model probabilities based on the external knowledge:

a. Determine the best *nbest* models for each number of variables using the leaps and bounds algorithm (*nbest* = 10 in our experiments).

b. For each selected model, compute its prior probability relative to the *w* candidates in the current BMA window using Equation (5).

c. Remove the *w* candidate regulators with posterior inclusion probability Pr(*β*_*gr*_ ≠ 0 | *D*) <5%.

5. Fill the *w*-candidate BMA window with those not considered yet in the shortlist.

6. Repeat steps 4–5 until all the *p* candidates in the shortlist have been processed.

7. Compute the prior probability for all selected models relative to all the *p* shortlisted candidates using Equation (5).

8. Take the collection of all models selected at any iteration of BMA, and apply Occam’s window, reducing the set of models.

9. Compute the posterior inclusion probability for each candidate regulator using the set of selected models, and infer candidates associated with a posterior probability exceeding a pre-specified threshold (50%) to be regulators for target gene *g*.

External knowledge is used in the following ways:

1. All the candidate regulators are ranked according to their regulatory potentials, which were predicted using the available external data sources at the supervised learning stage.

2. Model selection is performed by comparing models against each other based on their posterior odds. As shown by Equation (6), the posterior odds is proportional to a product of the integrated likelihood and the prior odds. The prior probability and, therefore, the prior odds, of a candidate model are formulated as a function of regulatory potentials.

3. The posterior inclusion probability of each candidate regulator, from which inference is made about the presence or absence of a regulatory relationship, is positively related to its regulatory potential. As shown in Equation (5), a factor of *π*_*gr*_ is contributed to each model in which the candidate *g* is included. Otherwise, a factor of 1- *π*_*gr*_ is contributed to each model.

## Abbreviations

BMA: Bayesian Model Averaging; iBMA: Iterative Bayesian Model Averaging; LAR: Least angle regression; LASSO: Least absolute shrinkage and selection operator; TF: Transcription factor.

## Competing interests

The authors declare that they have no competing interest.

## Author contributions

KL and AER developed the methodology. KL implemented the methods. KL and KYY analyzed the data. KMD performed and JZ, EES, REB designed the experiments. AER and KYY conceived the study. KL, AER and KYY wrote the manuscript. All authors read, edited and approved the final manuscript.

## Supplementary Material

Additional file 1 Supplementary figures. Click here for file

Additional file 2 Supplementary tables. Click here for file

Additional file 3 Text containing supplementary materials and methods. Click here for file
