# Supplementary material for: Integrating external biological knowledge in the construction of regulatory networks from time-series expression data
Source: BMC Syst Biol. 2012 Aug 16;6:101. doi: 10.1186/1752-0509-6-101 (PMC3465231; doi:10.1186/1752-0509-6-101)

Integrating External Biological Knowledge in the Construction of Regulatory Networks from Time-series Expression Data: Additional file 1

Kenneth Lo, Adrian E. Raftery, Kenneth M. Dombek, Jun Zhu, Eric E. Schadt, Roger E. Bumgarner, Ka Yee Yeung

SUPPLEMENTARY FIGURES

Figure S1. **Assessment criteria for recovery of regulatory relationships documented in the Yeastract database.**
The true positive rate (TPR), the number of misclassified cases, and the O/E ratio are defined in terms of entries of a 2×2 contingency table.

TF-gene pairs in the inferred network?	TF-gene pairs documented in Yeastract?		
		Yes	No
	Yes	TP	FP
	No	FN	TN

TPR (%) = TP / (TP + FP) x 100
misclassified cases = FP + FN
O/E ratio = TP / [(TP + FP) (TP + FN) / (TP + FP + FN + TN)]

Figure S2. **Incremental merit of the three correction strategies in the extension of the supervised framework.**

(a) Before and after the adjustment for the sampling bias regarding positive and negative training cases.

(b) Before and after the imputation of missing ChIP-chip values and truncation of extreme values.

(a)

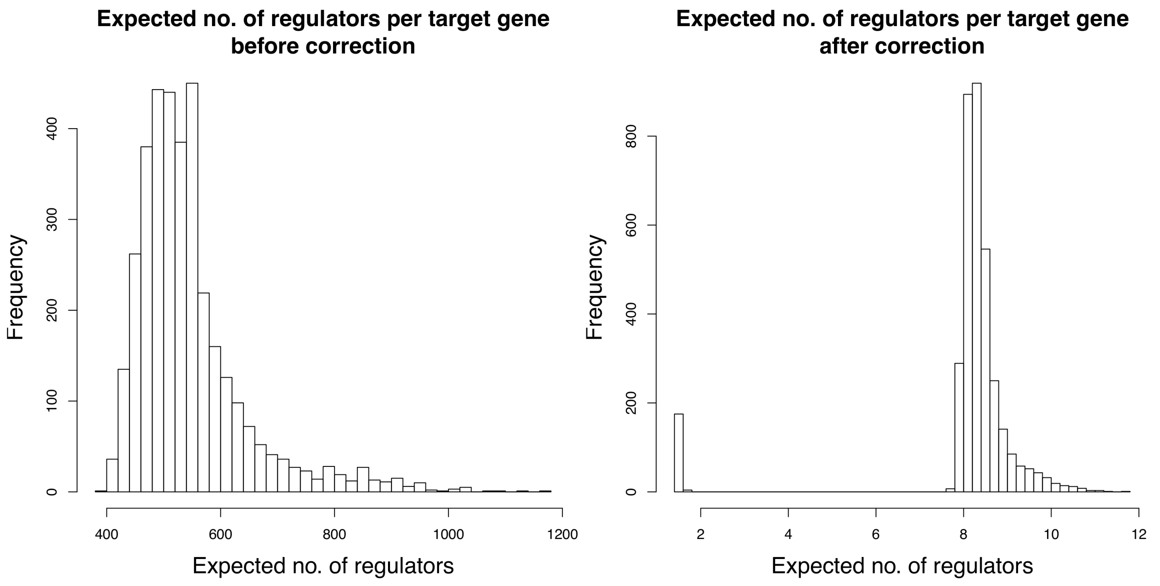

(b)

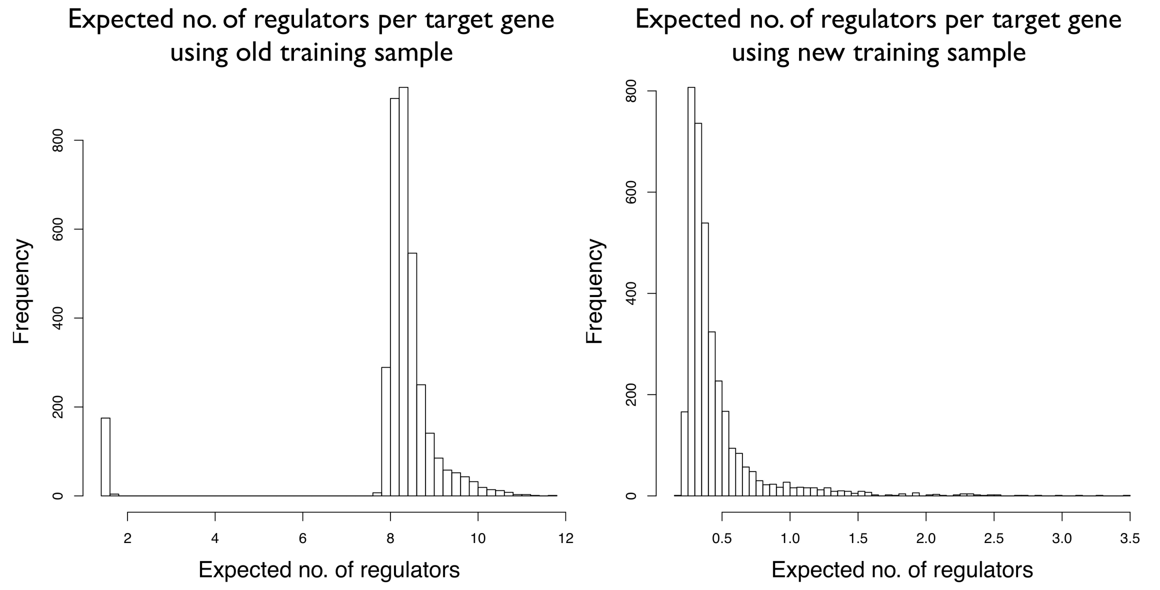

Figure S3. **Plots to compare the networks constructed by iBMA-prior with τ=0.00046 and τ=0.00083 respectively.**
A. Histogram of the ratio of the number of regulators inferred by iBMA-prior with τ=0.00046 to iBMA-prior with τ=0.00083; **B.** Fraction of common regulators to the intersection of regulators inferred by either setting.

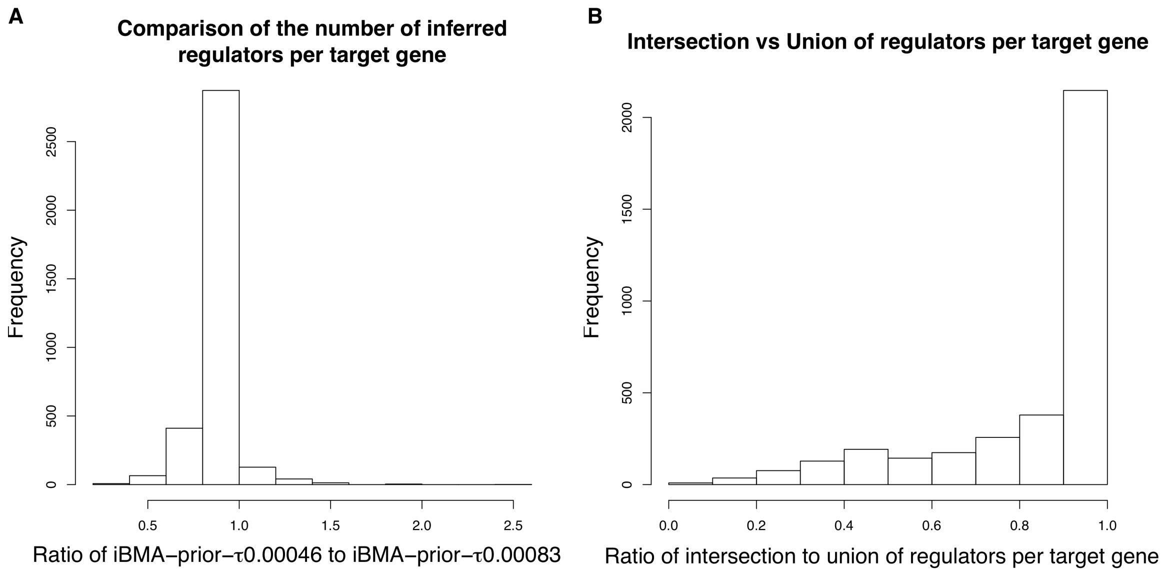

Figure S4. **Precision-Recall (PR) curve for iBMA-prior with τ=0.00046 on the time-series yeast gene expression data.** Precision (or TPR) is defined as TP/(TP+FP) and recall (or sensitivity) is defined as TP/(TP+FN) using the notations in Supplementary Figure S1. The two vertical lines in cyan show the range of recall obtained by thresholding the top ranked edges (0.0005 to 0.0441). The curve outside this range was obtained by interpolation.

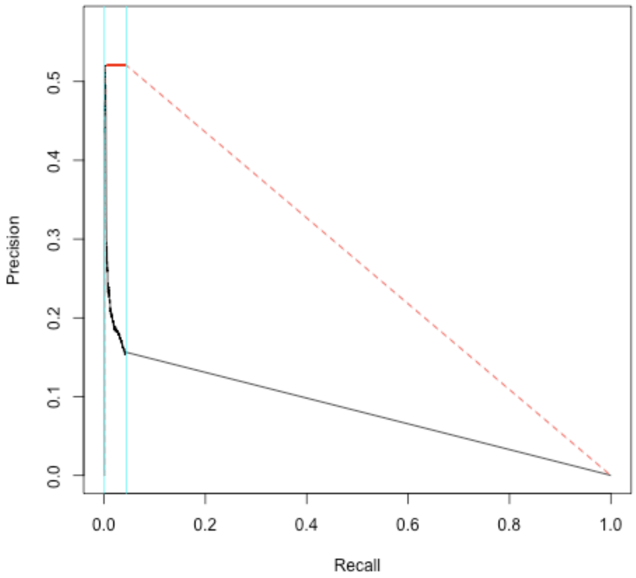

Supplement: Additional file 1 — Supplementary figures. [file 1752-0509-6-101-S1.pdf]
