# Supplementary material for: Integrating external biological knowledge in the construction of regulatory networks from time-series expression data
Source: BMC Syst Biol. 2012 Aug 16;6:101. doi: 10.1186/1752-0509-6-101 (PMC3465231; doi:10.1186/1752-0509-6-101)
Supplement: Additional file 2 — Supplementary tables. [file 1752-0509-6-101-S2.pdf]

Integrating External Biological Knowledge in the Construction of Regulatory Networks from Time-series Expression Data

Additional file 2: SUPPLEMENTARY TABLES

Kenneth Lo, Adrian E. Raftery, Kenneth M. Dombek, Jun Zhu, Eric E. Schadt, Roger E. Bumgarner, Ka Yee Yeung

Table S1. **Detailed assessment result for different network construction methods on the time-series yeast gene expression data.**

Method	Data used	Network size	*p*-value of χ^2 test[a]	TPR (%)[b]	# mis-class.[c]	O/E[d]	TP	FP	FN	TN
iBMA-prior - τ0.00046	Gene expression + external data	21951	<1.00E-320	18.00	19282	4.11	593	2702	16580	372237
iBMA-prior - τ0.00083	Gene expression + external data	23239	<1.00E-320	17.85	19421	4.08	624	2872	16549	372067
iBMA-shortlist	Gene expression + external data	65122	1.68E-111	9.98	22485	2.28	662	5974	16511	368965
LASSO-shortlist	Gene expression + external data	255293	<1.00E-320	11.07	46482	2.53	4169	33478	13004	341461
LAR-shortlist	Gene expression + external data	242495	<1.00E-320	11.28	44765	2.57	4017	31609	13156	343330
iBMA-size- τ0.00046	Gene expression data only	17202	5.75E-56	16.84	17622	3.84	114	563	17059	374376
iBMA-noprior	Gene expression data only	63026	1.75E-23	8.85	18903	2.02	186	1916	16987	373023
LASSO-noprior	Gene expression data only	564321	8.24E-27	5.96	33487	1.36	1103	17417	16070	357522
LAR-noprior	Gene expression data only	194687	1.38E-40	7.71	22777	1.76	511	6115	16662	368824

[a] The *p*-value of Pearson's chi-square test measures the strength of association between an inferred network and the Yeastract database.
[b] True positive rate (TPR) is defined as the proportion of inferred regulatory relationships that are documented in Yeastract.
[c] The number of misclassified cases is the sum of false positives (FP) and false negatives (FN).
[d] The O/E ratio is the number of folds the observed number of recovered relationships (i.e., TP) in excess of the expected count of recovery by chance.

Networks inferred using iBMA-prior, iBMA-size, iBMA-noprior and iBMA-shortlist were thresholded at posterior probability >= 50%.

Table S2. **Assessment results for iBMA over different posterior probability thresholds on the time-series yeast gene expression data.** As we vary the posterior probability thresholds (50%, 75%, 90%, 100%), iBMA-prior and iBMA-size out-perform iBMA-shortlist and iBMA-noprior.

Method	posterior probability	Network size	TPR (%)	O/E	TP	FP	FN	TN
iBMA-prior - τ0.00046	50%	21951	18	4.11	593	2702	16580	372237
iBMA-shortlist	50%	65122	9.98	2.28	662	5974	16511	368965
iBMA-size-τ0.00046	50%	17202	16.84	3.84	114	563	17059	374376
iBMA-noprior	50%	63026	8.85	2.02	186	1916	16987	373023
iBMA-prior - τ0.00046	75%	19390	18.28%	4.17	536	2396	16637	372543
iBMA-shortlist	75%	57586	10.37%	2.37	615	5314	16558	369625
iBMA-size-τ0.00046	75%	15058	17.96%	4.10	109	498	17064	374441
iBMA-noprior	75%	55380	9.22%	2.11	172	1693	17001	373246
iBMA-prior - τ0.00046	90%	17434	18.91%	4.32	502	2153	16671	372786
iBMA-shortlist	90%	51277	10.79%	2.46	576	4762	16597	370177
iBMA-size-τ0.00046	90%	13489	18.74%	4.28	104	451	17069	374488
iBMA-noprior	90%	49161	9.80%	2.24	165	1518	17008	373421
iBMA-prior - τ0.00046	100%	15524	19.31%	4.41	456	1906	16717	373033
iBMA-shortlist	100%	40229	11.35%	2.59	492	3841	16681	371098
iBMA-size-τ0.00046	100%	12080	19.84%	4.53	102	412	17071	374527
iBMA-noprior	100%	38418	10.25%	2.34	138	1208	17035	373731

True positive rate (TPR) is defined as the proportion of inferred regulatory relationships that are documented in Yeastract.

The O/E ratio is the number of folds the observed number of recovered relationships (i.e., TP) in excess of the expected count of recovery by chance.

Table S3. **Estimated regression coefficient and the posterior probability for each external data type at the revised supervised learning stage.**

External data type	Category[a]	Estimated coefficient	Posterior prob. (%)
Correlation in environmental stress data [9]	P	1.73	100
Correlation in Rosetta compendium data [10]	P	2.26	100
Correlation in Stanford gene expression data [11]	P	-2.23	100
Strength of binding evidence in ChIP-chip experiments [12]	P	-0.92	100
# GO [13] terms shared in common	P	0.20	97
Existence of known cis-regulation	R	0	0
Existence of known regulatory role [14]	R	3.23	100
# SNPs in regulator [15]	R	0	0
# non-synonymous coding SNPs [15]	R	0.87	100
# synonymous coding SNPs [15]	R	0	0
# SNPs in untranslated region at 5' end [15]	R	0.06	24
# SNPs in untranslated region at 3' end [15]	R	0	0
# SNPs in upstream promoter region [15]	R	-0.01	18
# SNPs in downstream promoter region [15]	R	0	0
Amino acid score [16]	R	-0.30	100

[a] Each external data type is classified into two categories: information about pairwise relationship between a candidate regulator and a target gene (P), and information about the candidate regulator only (R).

Table S4. **Summary of the assessment result for iBMA-size relative to different estimates of network density on the time-series gene expression data in yeast.**

Method	Network size	*p*-value of χ^2 test	TPR (%)	# misclass.	O/E	TP	FP	FN	TN
iBMA-size-τ0.00046	17202	5.75E-56	16.84	17622	3.84	114	563	17059	374376
iBMA-size-τ0.001	18195	2.05E-49	15.80	17658	3.61	112	597	17061	374342
iBMA-size-τ0.002	19287	6.07E-44	14.69	17716	3.36	113	656	17060	374283
iBMA-size-τ0.003	19992	8.69E-46	14.75	17732	3.37	117	676	17056	374263
iBMA-size-τ0.01	22559	1.67E-50	14.78	17788	3.37	129	744	17044	374195

Table S5. **Area under the Precision-Recall curve (PRC) for iBMA-based methods on the time-series gene expression data in yeast.** Precision (or TPR) is defined as TP/(TP+FP) and recall (or sensitivity) is defined as TP/(TP+FN) using the notations in Supplementary Figure S1.

**Method**	**Network size**	**Area under PRC**
iBMA-prior - τ0.00046	21951	0.0842
iBMA-shortlist	65122	0.0529
iBMA-size- τ0.00046	17202	0.0713
iBMA-noprior	63026	0.0395
