# Supplementary material for: Integrating external biological knowledge in the construction of regulatory networks from time-series expression data
Source: BMC Syst Biol. 2012 Aug 16;6:101. doi: 10.1186/1752-0509-6-101 (PMC3465231; doi:10.1186/1752-0509-6-101)
Supplement: Additional file 3 — Text containing supplementary materials and methods. [file 1752-0509-6-101-S3.pdf]

Integrating External Biological Knowledge in the Construction of Regulatory Networks from Time-series Expression Data: Additional file 3

Kenneth Lo, Adrian E. Raftery, Kenneth M. Dombek, Jun Zhu, Eric E. Schadt, Roger E. Bumgarner, Ka Yee Yeung

SUPPLEMENTARY MATERIALS AND METHODS

Implementation

iBMA-prior was applied to the yeast time-series gene expression data set by first computing the regulatory potentials at the supervised learning stage, using the same external knowledge as in our previous work reported in Yeung et al. [1]. To address the issue of sampling bias towards positive cases in the training data, we applied the adjustment with τ=0.00046. For each target gene, we shortlisted the top p=100 candidate regulators with the highest regulatory potentials. We then applied iBMA-prior to the time-series gene expression data. At each run of BMA, a window size of w=30 was maintained, and the best *nbest*=10 models as per each model size were returned from leaps and bounds. At the end of iBMA, the Occam's window was applied to discard models with posterior odds of less than 1/20 against the best model (i.e., the one with the highest posterior probability). Out of the p=100 candidates, we inferred those with a posterior inclusion probability ≥50% to be regulators of the target gene. We adopted the same values of parameters from previous applications of BMA [2-5]. We used the above strategy to infer regulators for each of the 3556 genes in the data set. Our current implementation in R took about 2 hours on 15 3.06GHz Intel Xeon processor each with 1GB of RAM to complete the analysis.

LASSO [6, 7] and LAR [8] are popular variable selection methods, and have been shown to return sparse solutions. Therefore, we included them in the analysis and compared our BMA-based methods to them. We implemented LASSO with the glmnet R package, while LAR was run using the lars R package. For both approaches, we performed 10-fold cross-validation to determine the optimal level of shrinkage that minimized the mean-squared error. In order to properly compare them with BMA-based approaches that utilized external biological knowledge, we also extended LASSO and LAR respectively to take external knowledge into consideration. Explicitly, preceding the applications of regular LASSO and LAR, we applied the results from the supervised learning stage detailed in the *Materials and Methods* section of the main text, and shortlisted p=100 candidate regulators for each gene.

Robustness against Different Estimates of Network Density

At the revised supervised learning stage, we adjusted for the sampling bias towards positive cases in the training sample with the aid of a density estimate. In the main text, we derived from the exponential distribution reported in Guelzim et al. [9], and set the density parameter τ = 2.76/6000 = 0.00046, where 2.76 is the average number of regulators per target gene. To show that the assessment results of our networks are robust with respect to this density parameter τ, we tested the setting τ = 5/6000 = 0.00083, where 5 is the $90th$ percentile of the aforementioned distribution. As shown in Additional file 2: Table S2, the assessment result was comparable to those two settings.

In addition, we zoomed into target genes individually to examine the level of agreement on the set of regulators with respect to the two network density estimates. The two settings led to a similar number of regulators inferred for each target gene, suggested by the histogram displayed in Additional file 1: Figure S3(a) that peaked around the ratio of one. This finding was consistent with the close network sizes reported in Additional file 2: Table S2. Additional file 1: Figure S3(b) shows that >80% of the inferred regulators were found in both networks for the majority of target genes. This high degree of concordance in the set of inferred regulators empirically justified that the proposed network inference strategy was relatively robust against different estimates of network density, an attribute of the underlying network that is rather difficult to be precisely estimated.

We also examined how these assessment results would vary with different network density estimates as input to the BMA-based network construction methods when no external data sources were used. Explicitly, we studied the performance of iBMA-size with respect to network density parameters τ=0.001, 0.002, 0.003 and 0.01. In iBMA-size, we applied iBMA to the time-series expression data with model size priors without using any information from the external data sources. Again, we compared each network constructed against the Yeastract database to assess its concordance with documented regulatory relationships. Additional file 2: Table S4 summarizes the assessment result with respect to different estimates of network density. Here we also included the result for τ=0.00046 that was reported in Additional file 2: Table S2 for reference. As expected, we observed an increasing trend in network size with larger network density parameters. On the contrary, for both the TPR and the O/E ratio, an overall, slightly decreasing trend was recorded; the best performance was observed at τ=0.00046, the original network density estimate derived from the empirical exponential distribution determined in previous literature [9]. In general, although a trend relative to the different assessment criteria was observed, the changes were, however, relatively subtle compared with the significant change in the network density specified. For instance, the highest level of network density attempted, τ=0.01, was >20 times of the original density of τ=0.00046. In spite of this inflated network density, the size of the inferred network only moderately increased to 22559 or 30%, and was accompanied by a small drop in TPR of 2%. This finding suggests that the impact of an imprecise estimate of network density on network inference would be properly attenuated by other available information.

Challenges of assessment

The main challenge of assessing networks inferred from real data is the lack of a complete and objective gold standard. In this work, we adopted the literature curated regulatory relationships in the Yeastract database [10]. The first issue is that our gold standard is not necessarily complete. In other words, our inferred networks might recover new true regulatory relationships that are not reported in the literature, and these new relationships would be incorrectly classified as false positives (FP, using the notations in Additional file 1: Figure S1). The second issue is that we do not know the true negatives, i.e., there are no databases documenting that a given TF does not regulate a certain gene. Due to these challenges, when we computed the 2 x 2 contingency table, we only considered the edges spanned by the set of TFs and genes covered by Yeastract. In the 3556-gene filtered time series gene expression data subset, the Yeastract documented regulatory relationships cover 127 TFs and 3092 genes. Using the results of iBMA-prior with τ=0.00046 as an example, there are 3295 edges in the inferred network spanned by these 127 TFs and 3092 genes. Using the notations in Additional file 1: Figure S1, FP = 3295 – TP = 3295 – 593 = 2702. There are a total of 17173 distinct TF-gene relationships from Yeastract in this filtered 3556-gene subset, so FN = 17173 – 593 = 16580. Note that we have subtracted the 572 hard-coded TF-gene relationships from the gold standard. Therefore, TN = 127 * 3092 – 572 – TP – FP – FN = 372237.

In the main text, we focused on inferred networks thresholded at a posterior probability of 50% (Tables 2-4). Additional file 2: Table S2 shows that iBMA-prior and iBMA-size out-perform iBMA-shortlist and iBMA-noprior as we vary the posterior probability thresholds. In addition, we defined precision = TPR = TP/(TP+FP), and recall = sensitivity = TP/(TP+FN) using the notations in Additional file 1: Figure S1. Additional file 2: Table S2 shows that even at posterior probability threshold of 100%, iBMA-prior still yields 15,524 edges. In order to rank the edges and break ties with the same posterior probability, we applied linear regression to the candidate regulators inferred from iBMA-prior and computed the t-statistics associated with the regression coefficients for each gene. We rank all edges in an inferred network first in descending order of the posterior probability from iBMA-prior, and second in descending order of the absolute value of the t-statistics from linear regression. We then computed precision and recall for the top ranked E edges, where E = 100, 200, 300, …, 15,524, and plotted the precision-recall curve in Additional file 1: Figure S4. In Additional file 1: Figure S4, the range of recall is between 0.0005 and 0.0441, the range of precision is 0.1552 to 0.8182, and the area under the curve is 0.0842. We repeated the calculation of precision and recall for iBMA-size (τ=0.00046), iBMA-shortlist and iBMA-noprior, and the area under the curve for each method is shown in Additional file 2: Table S5. This assessment strategy shows the same trend as thresholding posterior probabilities at 50%: iBMA-prior and iBMA-size are superior to iBMA-shortlist and iBMA-noprior.

REFERENCES

1. Yeung KY, Dombek KM, Lo K, Mittler JE, Zhu J, Schadt EE, Bumgarner RE, Raftery AE: **Construction of regulatory networks using expression time-series data of a genotyped population**. *Proc Natl Acad Sci U S A* 2011, **108**:19436-19441.

2. Madigan D, Raftery A: **Model selection and accounting for model uncertainty in graphical models using Occam's window**. *J Am Stat Assoc* 1994, **89**:1335-1346.
3. Raftery AE: **Bayesian model selection in social research (with discussion)**. *Sociol Methodol* 1995, **25**:111-193.
4. Yeung KY, Bumgarner RE, Raftery AE: **Bayesian model averaging: development of an improved multi-class, gene selection and classification tool for microarray data**. *Bioinformatics* 2005, **21**:2394-2402.
5. Yeung KY, Dombek KM, Lo K, Mittler JE, Bumgarner RE, Raftery AE: **Construction of regulatory networks using time series microarray data in genotyped yeast segregants.** *Under review* 2011.
6. Friedman J, Hastie T, Tibshirani R: **Regularization paths for generalized linear models via coordinate descent**. *J Stat Softw* 2010, **33**:1-22.
7. Tibshirani R: **Regression shrinkage and selection via the LASSO**. *J R Stat Soc Series B Stat Methodol* 1996, **58**:267-288.
8. Efron B, Hastie T, Johnstone I, Tibshirani R: **Least angle regression**. *Ann Stat* 2004, **32**:407-499.
9. Guelzim N, Bottani S, Bourgine P, Képès F: **Topological and causal structure of the yeast transcriptional regulatory network**. *Nat Genet* 2002, **31**:60-63.
10. Teixeira MC, Monteiro P, Jain P, Tenreiro S, Fernandes AR, Mira NP, Alenquer M, Freitas AT, Oliveira AL, Sa-Correia I: **The YEASTRACT database: a tool for the analysis of transcription regulatory associations in Saccharomyces cerevisiae**. *Nucleic Acids Res* 2006, **34**:D446-451.
